# DNNBrain: A Unifying Toolbox for Mapping Deep Neural Networks and Brains

**DOI:** 10.3389/fncom.2020.580632

**Published:** 2020-11-30

**Authors:** Xiayu Chen, Ming Zhou, Zhengxin Gong, Wei Xu, Xingyu Liu, Taicheng Huang, Zonglei Zhen, Jia Liu

**Affiliations:** ^1^Beijing Key Laboratory of Applied Experimental Psychology, Faculty of Psychology, Beijing Normal University, Beijing, China; ^2^State Key Laboratory of Cognitive Neuroscience and Learning, Beijing Normal University, Beijing, China

**Keywords:** deep neural network, brain imaging, neural representation, neural encoding and decoding, representational similarity analysis (RSA), feature visualization

## Abstract

Deep neural networks (DNNs) have attained human-level performance on dozens of challenging tasks via an end-to-end deep learning strategy. Deep learning allows data representations that have multiple levels of abstraction; however, it does not explicitly provide any insights into the internal operations of DNNs. Deep learning's success is appealing to neuroscientists not only as a method for applying DNNs to model biological neural systems but also as a means of adopting concepts and methods from cognitive neuroscience to understand the internal representations of DNNs. Although general deep learning frameworks, such as PyTorch and TensorFlow, could be used to allow such cross-disciplinary investigations, the use of these frameworks typically requires high-level programming expertise and comprehensive mathematical knowledge. A toolbox specifically designed as a mechanism for cognitive neuroscientists to map both DNNs and brains is urgently needed. Here, we present DNNBrain, a Python-based toolbox designed for exploring the internal representations of DNNs as well as brains. Through the integration of DNN software packages and well-established brain imaging tools, DNNBrain provides application programming and command line interfaces for a variety of research scenarios. These include extracting DNN activation, probing and visualizing DNN representations, and mapping DNN representations onto the brain. We expect that our toolbox will accelerate scientific research by both applying DNNs to model biological neural systems and utilizing paradigms of cognitive neuroscience to unveil the black box of DNNs.

## Introduction

Over the past decade, artificial intelligence (AI) has been able to make dramatic advances because of the rise of deep learning (DL) techniques. DL makes use of deep neural networks (DNNs) to model complex non-linear relationships and thus is able to solve real-life problems. A DNN often consists of an input layer, multiple hidden layers, and an output layer. Each layer generally implements some non-linear operations that transform the representation at one level into another representation at a more abstract level. In one particular example, deep convolutional neural network (DCNN) architecture stacks multiple convolutional layers hierarchically, inspired by the hierarchical organization of the primate ventral visual stream. A supervised learning algorithm is generally used to tune the parameters of the network to minimize errors between the network output and the target label in an end-to-end manner (LeCun et al., 1998; Rawat and Wang, 2017). As a result, DL is able to automatically discover multiple levels of representations that are needed for a given task (LeCun et al., 2015; Goodfellow et al., 2016). With this built-in architecture and learning from large external datasets, DCNNs have achieved human-level performance on a variety of challenging object (Krizhevsky et al., 2012; Simonyan and Zisserman, 2015; Szegedy et al., 2015; He et al., 2016) and speech recognition tasks (Hinton et al., 2012; Sainath et al., 2013; Hannun et al., 2014).

In addition to these achievements in engineering, DNNs provide a potentially rich interaction between studies on both biological and artificial information processing systems. On the one hand, DNNs offer the best models of biological intelligence to date (Cichy and Kaiser, 2019; Richards et al., 2019). In particular, good correspondence between DNNs and the visual systems has been identified (Yamins and DiCarlo, 2016; Kell and McDermott, 2019; Serre, 2019; Lindsay, 2020). First, DNNs exhibit behavioral patterns similar to those of human and non-human primate observers on some object recognition tasks (Jozwik et al., 2017; Rajalingham et al., 2018; King et al., 2019). Second, DCNNs appear to recapitulate the representation of visual information along the ventral stream. That is, early stages of the ventral visual stream (e.g., V1) are well-predicted by early layers of DNNs optimized for visual object recognition, whereas intermediate stages (e.g., V4) are best predicted by intermediate layers and late stages (e.g., IT) are best predicted by late layers (Khaligh-Razavi and Kriegeskorte, 2014; Yamins et al., 2014; Güçlü and van Gerven, 2015; Eickenberg et al., 2017). Finally, DNNs designated for object recognition spontaneously generate many well-known behavioral and neurophysiological signatures of cognitive phenomena such as shape tuning (Pospisil et al., 2018), numerosity (Nasr et al., 2019), and visual illusions (Watanabe et al., 2018). Thus, DNNs provide a new perspective to study the origin of intelligence. Indeed, neuroscientists have already used DNNs to model the primate visual system (Schrimpf et al., 2018; Lindsey et al., 2019; Lotter et al., 2020).

Alternatively, the end-to-end DL strategy makes DNN a black box, without any explanation of its internal representations. Experimental paradigms and theoretical approaches from cognitive neuroscience have significantly advanced our understanding of how DNNs work (Hasson and Nusbaum, 2019). First, concepts and hypotheses from cognitive neuroscience, such as sparse coding and modularity, provide a hands-on terminology to describe the internal operations of DNNs (Agrawal et al., 2014; Ritter et al., 2017). Second, a variety of methods of manipulating stimuli, such as stimulus degradation and simplification, have been used to characterize unit response dynamics (Baker et al., 2018; Geirhos et al., 2019). Finally, the rich data analysis techniques from cognitive neuroscience, such as ablation analysis (Morcos et al., 2018; Zhou et al., 2018), activation maximization (Nguyen et al., 2016), and representation similarity analysis (Khaligh-Razavi and Kriegeskorte, 2014; Jozwik et al., 2017), provide a powerful arsenal for exploring the computational mechanisms of DNNs.

Such a crosstalk between cognitive neuroscience and AI needs an integrated toolbox that meets the objectives of both fields. However, the most commonly used DL frameworks such as PyTorch^1^ and TensorFlow^2^ are developed for AI researchers. The use of these frameworks typically requires advanced programming expertise and comprehensive mathematical knowledge of DL. To our knowledge, there is no software package, specifically designed for both AI scientists and cognitive neuroscientists, that is able to interrogate DNNs and brains at the same time. Therefore, it would be of great value to have a unifying toolbox that maximally integrates DNN software packages and well-established brain mapping tools.

In this paper, we present DNNBrain, a Python-based toolbox specifically designed for exploring representations of both DNNs and brains. The toolbox has five major features.

- Versatility: DNNBrain supports a diverse range of applications for exploring DNN and brain representations. These include accessing DNN representations, building an encoding/decoding model for external stimuli, analyzing representational similarity between DNN and brain, transfer learning from pretrained models on study-specific stimuli, and visualizing DNN representations. Moreover, DNNBrain supports multiple modalities of input stimulus including image, audio, and video.
- Usability: DNNBrain provides a command line interface (CLI) and an application programming interface (API) for the user's convenience. At the application level, users can directly run commands to conduct typical representation analysis for both DNN and brain without any programming needed. At the programming level, all algorithms and computational pipelines are encapsulated into objects with high-level interface in the experimental design and data analysis language of neuroscientists. Users can easily program their own pipelines on these encapsulated algorithms objects.
- Transparent input/output (IO): DNNBrain transparently reads and writes multimodal neuroimaging data and multiple customized meta-data. As a result, DNNBrain spares users from the need to have specific knowledge about different data formats.
- Open source: DNNBrain is freely available in source. Users can access every detail of DNNBrain implementation. This improves the reproducibility of experimental results, leads to efficient debugging, and allows for accelerated scientific progress.
- Portability: DNNBrain, implemented in Python, runs on all major systems (e.g., Windows, Mac, and Linux). It is easy to set up, as it has no complicated dependencies on external libraries and packages.

As follows, we first introduce the functionalities of DNNBrain and then describe its framework (i.e., building blocks). Finally, with a typical application example, we demonstrate the versatility and usability of DNNBrain in characterizing both DNNs and brains as well as in examining the correspondences between DNNs and brains. The toolbox is freely available for download^3^ and complemented with an expandable online documentation.^4^

### Functionalities of DNNBrain

The primary aim of DNNBrain is to provide a framework that makes it easy to explore the internal representations of DNNs and brains, and the representational similarity between them. To do this, DNNBrain integrates a diverse range of tools such as encoding/decoding models to reveal stimuli or behavioral relevance of the representations, encoding/decoding models to map DNNs representations to those of brains, representational similarity analysis (RSA) between DNNs and brains, visualizing DNN representations, and transfer learning from pretrained models on study-specific stimuli.

#### Encoding and Decoding Model

**Figure 1:**
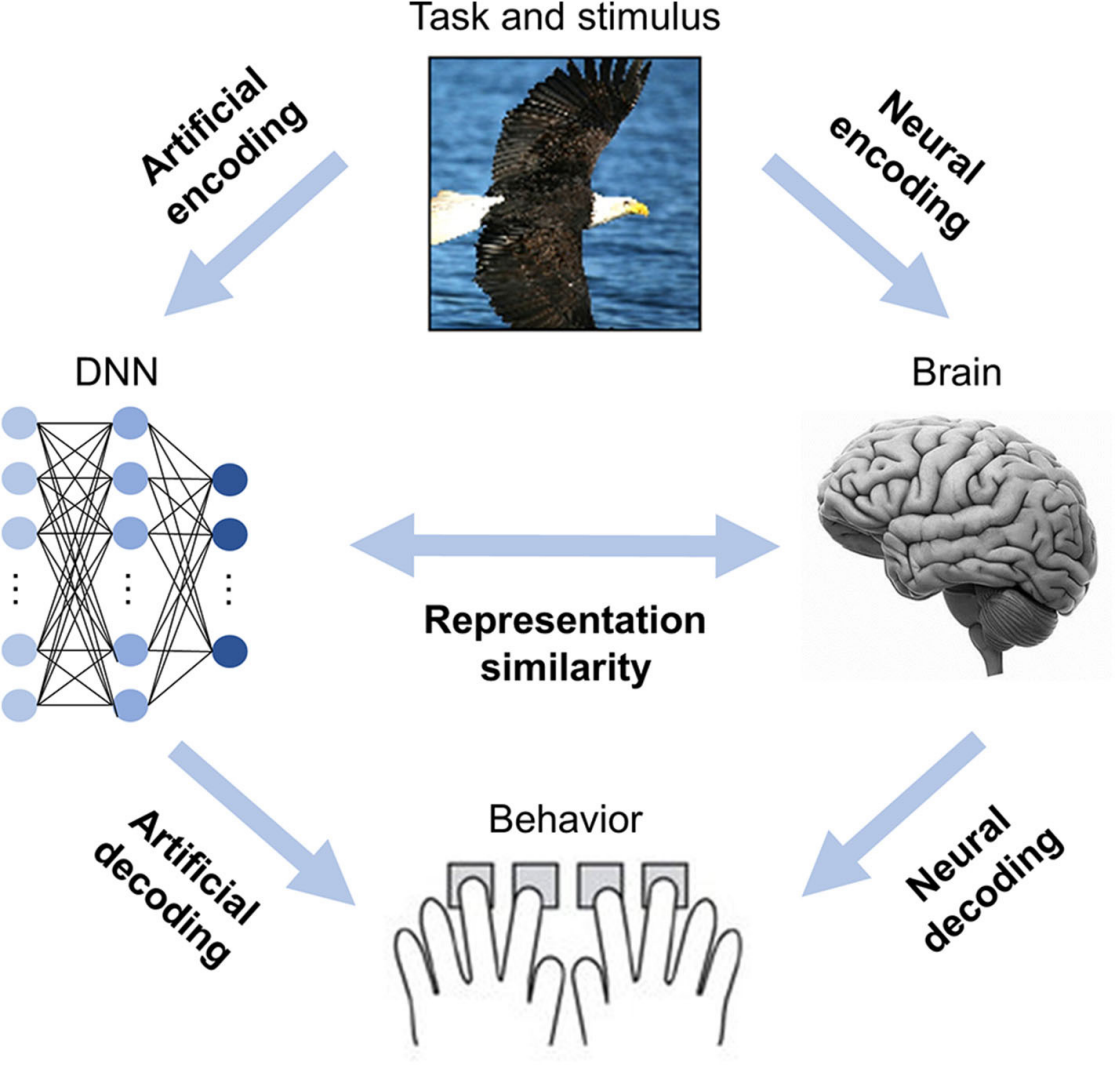
DNNBrain is designed as an integrated toolbox that characterizes artificial representations of DNNs and neural representations of brains. After stimuli are submitted to both DNNs and brains, the artificial neural activities, and the biological neural activities are acquired. By assembling the stimuli, the artificial activity data, and the biological neural activity data together with custom-designed auxiliary IO files, DNNBrain allows users to easily characterize, compare, and visualize representations of DNNs and brains.

Information processing in the brain and DNNs can generally be divided into two stages: (1) the neural code is generated from the stimuli (i.e., map stimuli to neural responses), and (2) the neural code is used to produce behavior (i.e., map neural responses to behavioral responses; Kriegeskorte and Douglas, 2019). In DNNBrain, neural (artificial) encoding models are implemented to do the former, whereas neural (artificial) decoding models are used for the latter (Figure 1).

**Figure 2:**
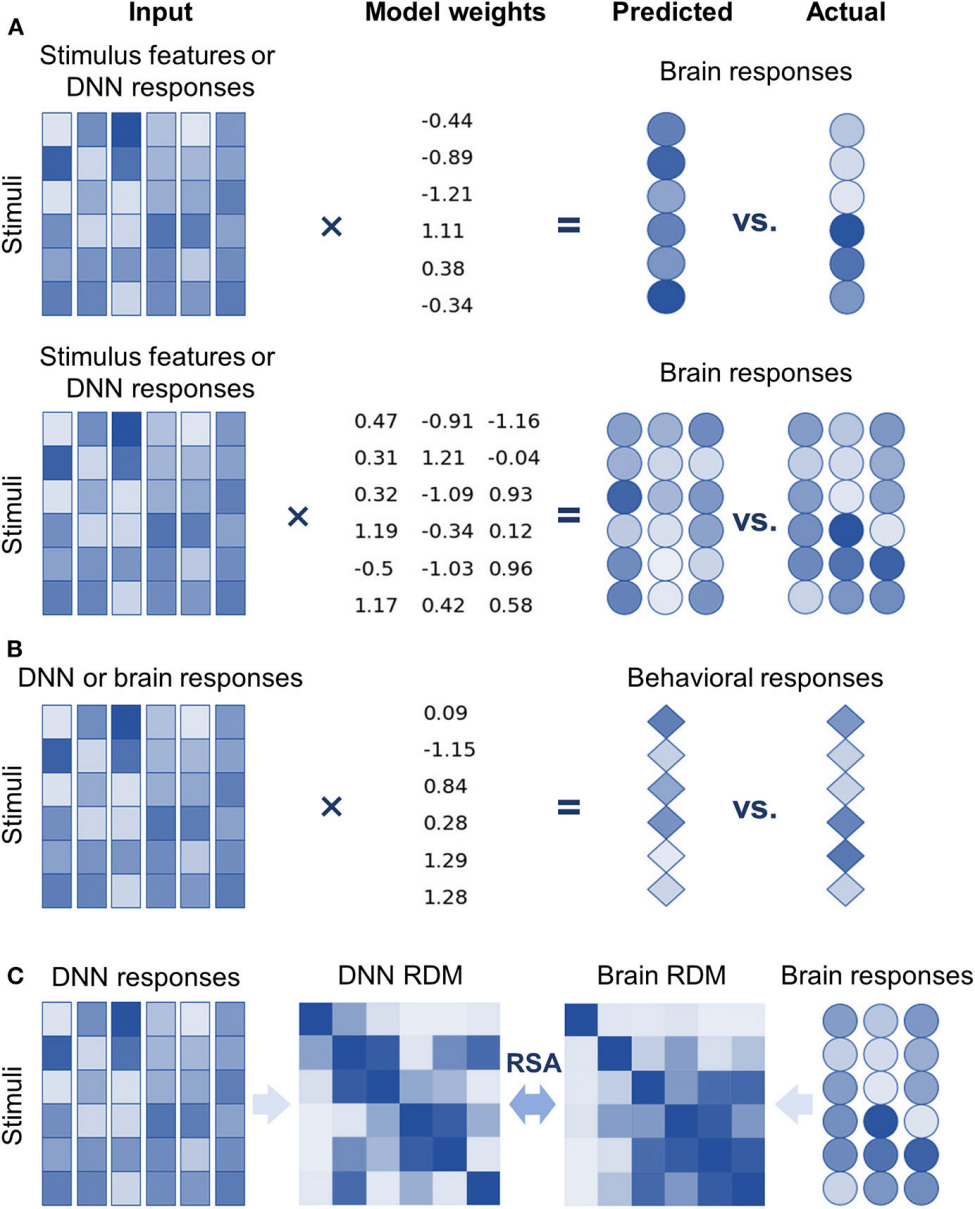
DNNBrain provides multiple approaches to explore internal representations of DNNs and the brain, and the representational similarities between them. **(A)** Top: univariate linear encoding models find optimal linear combinations of multiple stimulus features (or DNN responses) to predict the response of a neuron/voxel. Bottom: multivariate linear models search optimal linear combinations of multiple stimulus features (or DNN responses) to predict the responses from multiple neurons/voxels by maximizing their covariance. **(B)** In the opposite direction of encoding models, linear decoding models find optimal linear combinations of neural responses (or DNN responses) to predict behavior responses. **(C)** Representational similarity analysis evaluates the similarity of two representations by comparing representational dissimilarity matrices obtained from them.

Encoding models are implemented as linear models because the manner in which features of stimuli are represented in an explicit format by a neuron/voxel is a primary concern of neuroscientists (Yamins et al., 2014; Wen et al., 2018). Two kinds of linear models were introduced into DNNBrain to support encoding models (Figure 2A). First, univariate linear models (e.g., GLM, ridge, and lasso regression) were adopted to find linear combinations of stimuli features to predict the response of a neuron/voxel (Naselaris et al., 2011). The univariate encoding model describes how information is encoded in the activity of the individual neuron/voxel; however, it ignores interactions between different neurons/voxels. Second, multivariate partial least squares (PLS) linear models were introduced to find linear relations in two sets of multivariate variables (i.e., stimulus features and neural responses) by maximizing covariance of the transformed variables (Bilenko and Gallant, 2016; O'Connell and Chun, 2018). PLS models the covariance structures of stimuli features and neural responses, and thus provides information on how individual features and their interactions contribute to predicting responses from multiple neurons/voxels. Decoding models, which predict behavioral responses based on neural responses, work in the opposite direction of encoding models. Therefore, univariate linear models used for encoding models can serve as decoding models by simply exchanging response variables for predictor variables of the encoding models (Figure 2B).

DNNBrain uses cross-validation (CV) techniques (e.g., k-fold and leave-one-out CV) to evaluate the generalization performance of encoding/decoding models. The CV techniques divide a dataset into several non-overlapping subsets. Each subset is held back in turn as the test set, whereas all other subsets are collectively used as a training dataset. The accuracy (i.e., the fraction of correct predictions) and explained variance are generally used to measure performance for classification- and regression-based encoding/decoding models, respectively. Permutation testing is utilized to test the significance of the model performance. The null distribution is generated by deriving the performance measure multiple times using original data samples, but with permuted targets.

#### Analyzing Representational Similarity

Another focus of DNNBrain is to provide tools to examine representational similarities between DNNs and brains (i.e., describe the relationships between neural responses from DNNs and those from brains) (Figure 1). First, encoding models can be used to examine the representational similarity between DNNs and brains if internal representations of DNNs are considered as extracted features of external stimuli (Figure 2A). Second, representational similarity analysis (RSA) was implemented in DNNBrain to evaluate the similarity between two representations (Kriegeskorte et al., 2008) (Figure 2C). RSA differs from encoding/decoding models, which measure the representational similarity between DNNs and brains by examining how brain responses could be directly predicted from DNN responses, or vice versa. In contrast, RSA utilizes pairwise comparison of stimuli in representation space to characterize their representation. Representational dissimilarity, which is often calculated as Euclidean distance or correlation distance between two multivariate response patterns, is first created for every pair of stimuli or conditions, and then summarized in a representational dissimilarity matrix (RDM) which characterizes the geometry of the set of points in the multivariate response space. Finally, the correlation between RDMs from DNNs and brains is calculated to measure their representational similarity. Multiple correlation metrics are supported by DNNBrain including the Pearson correlation, Kendall's tau correlation, and Spearman's correlation. Permutation tests were integrated in DNNBrain to estimate significance of the representational similarity between DNNs and brains. The permutation test randomizes the stimulus labels multiple times to generate the null distribution.

#### Transfer Learning From Pretrained Models on Study-Specific Stimuli

Training a DNN from scratch often requires a large amount of computational demand that results in significant time and energy costs. Moreover, there usually is not enough existing data available to train a DNN *de novo*. Fortunately, it turns out that representations from pretrained DNNs on large datasets (e.g., ImageNet) often work well for related new tasks. Therefore, instead of training a DNN from scratch, it can be trained to solve a new task by fine-tuning the weights of a pretrained model using just a very few training examples. This is known as transfer learning. Clearly, transfer learning is of great value in the study of representational similarities between DNNs and brains because it is often not possible to collect large-scale neural datasets. DNNBrain provides a set of utilities that assists users in transfer learning from pretrained DNNs on their study-specific dataset. Users can easily specify which target layers/channels to be fine-tuned and customize the new task layers.

#### Visualizing Features From DNNs

DNNs are a kind of complex non-linear transformation that does not provide explicit explanation of their internal workings. Identifying the relevant features that contribute most to the responses of an artificial neuron is central to the understanding of precisely what each neuron has learned (Montavon et al., 2018; Nguyen et al., 2019). Three approaches have been implemented in DNNBrain to assist users in examination of the stimulus features that an artificial neuron prefers. The first approach is known as *top stimulus discovering*. The top images with the highest activations for a specific neuron (or unit) are identified from a large image collection (Zeiler and Fergus, 2014; Yosinski et al., 2015). The second approach, known as *saliency mapping*, computes gradients on the input images relative to the target unit, utilizing a backpropagation algorithm. It highlights pixels of the image that change the unit's activation most when its value changes (Simonyan et al., 2014; Springenberg et al., 2015). The third approach is termed *optimal stimulus synthesizing*. This approach synthesizes the visual stimulus from initial random noise, guided by increasing activation of the target neuron (Erhan et al., 2009; Nguyen et al., 2016).

#### Other Utilities Provided by DNNBrain

In addition to the functionalities described previously, DNNBrain provides additional flexible pipelines for neuroscience-orientated analysis of DNNs. These include ablation analysis of individual units (Morcos et al., 2018; Zhou et al., 2018) and estimation of the empirical receptive field of a unit (Zhou et al., 2014). It also comes with a variety of utilities, such as image processing tools used for converting different data structures (e.g., PyTorch tensor, NumPy array, and PIL image objects), translating and cropping images, and more. Details can be found on the DNNBrain documentation page.^4^

### Implementation of DNNBrain

**Figure 3:**
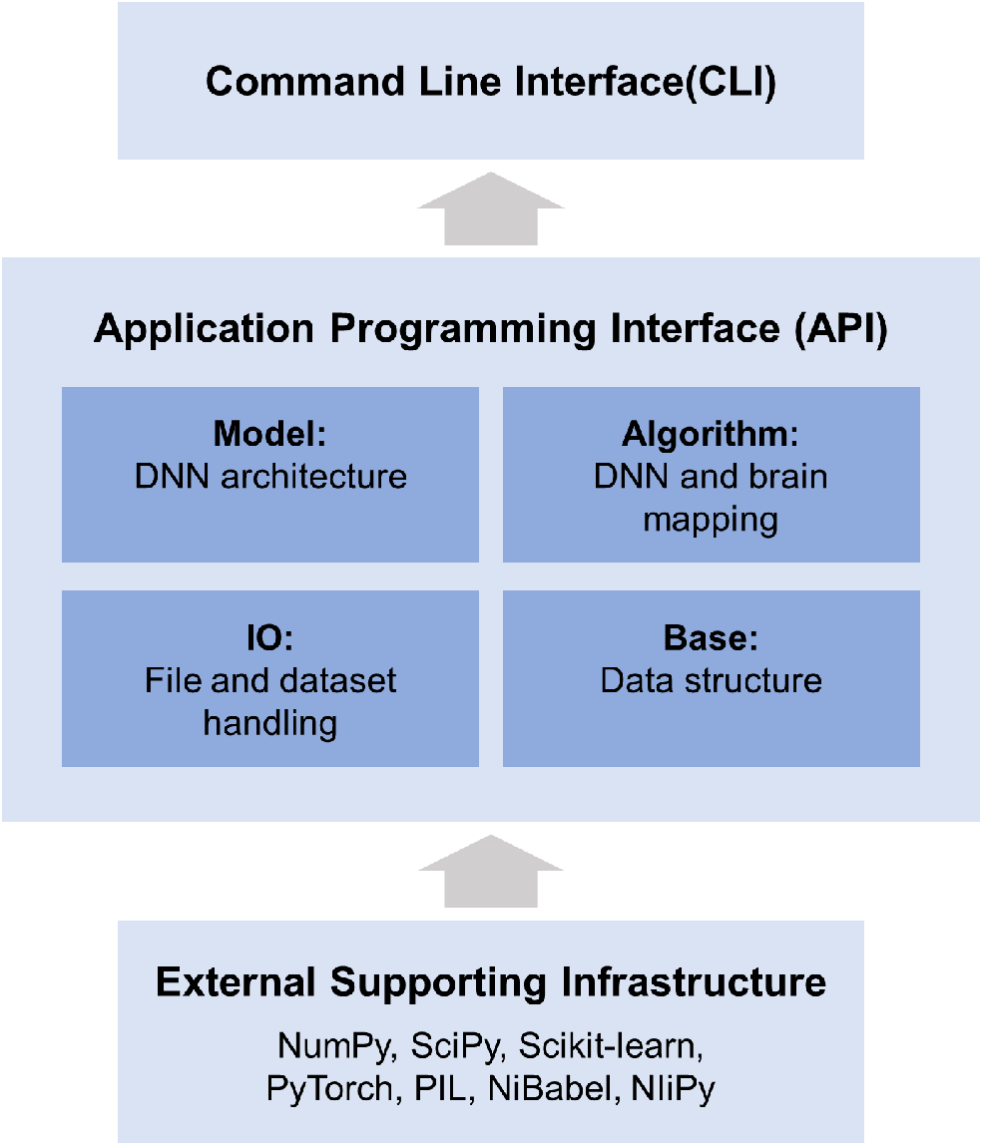
DNNBrain is a modular framework which consists of four modules: IO, Base, Model, and Algorithm. The IO module provides facilities for managing file-related input and output operations. The Base module defines base level classes for array computing and data transforming. The Model module holds a variety of DNN models. The Algorithm module defines various algorithms for exploring DNNs and the brain. All modules provide user-friendly APIs. A set of CLIs was developed for a variety of research scenarios.

DNNBrain is a modular Python toolbox that consists of four modules: IO, Base, Model, and Algorithm (Figure 3). The Python language was selected for DNNBrain because it provides an ideal environment for the research on DNNs and brains. First, Python is currently the most commonly used programming language for scientific computing. Many excellent Python libraries have been developed for scientific computing. The libraries used in the DNNBrain are as follows: NumPy for numerical computation,^5^ SciPy for general-purpose scientific computing,^6^ scikit-learn for machine learning,^7^ and Python imaging library (PIL) for image processing.^8^ Second, Python is increasingly used in the field of brain imaging. Many Python libraries for brain imaging data analysis have been developed such as NiPy^9^ (Millman and Brett, 2007) and fMRIPrep^10^ (Esteban et al., 2019). Finally, Python is the most popular language in the field of DL. Python is well-supported by the two most popular DNN libraries (i.e., PyTorch^1^ and TensorFlow^2^).

Supported by a large variety of existing software packages, DNNBrain was designed with a high-level API in the domain language of cognitive neuroscience. All algorithms and computational pipelines are encapsulated into classes in an object-oriented programming manner. All modules provide user-friendly APIs. On these APIs, a set of CLIs was developed for a variety of research scenarios.

Of note, neuroimaging data preprocessing pipelines are not included in DNNBrain. The data need to be preprocessed before they are input into DNNBrain. This separation between the DNNBrain representation analysis pipeline and the data preprocessing pipeline provides users with maximum flexibility to utilize different neuroimaging toolboxes to preprocess their data.

#### IO Module: Organizing Datasets in DNNBrain

DNNBrain introduces auxiliary file formats to handle various types of scientific data and supporting metadata. These include stimulus files, DNN mask files, and DNN activation files. With these file formats, users can easily organize their inputs and outputs. The stimulus file is a comma separated values (CSV) text file designed to configure stimulus information including the stimulus type (image, audio, and video), stimulus directory, stimulus ID, stimulus duration, stimulus conditions, and other possible stimulus attributes. The DNN mask file is also a CSV text file designed for users to specify channels and units of interest when analyzing DNNs. Both the stimulus file and the DNN mask file can be easily configured with a text editor. The DNN activation file is a HDF5 (Hierarchical Data Format) file in which activation values from specified channels are stored. In addition, DNNBrain uses NiBabel^11^ to access brain imaging files. Almost all common MRI file formats are supported, including GIFTI, NIfTI, CIFTI, and MGH.

#### Base Module: Defining the Basic Data Structure

The base module defines base level objects for data structure and data transformations. Specifically, a set of objects is defined to organize either data from the input stimulus or the output activation data from the DNN. The data objects were designed to be as simple as possible, while retaining necessary information for further representation analysis. The stimulus object contains stimulus paths and associated attributes (e.g., category label), which are read from stimulus files. The activation object holds DNN activation patterns and associated location information (e.g., layer, channel, and unit). Aside from these data objects, several encoding/decoding models were developed, including popular classification and regression models such as generalized linear models, logistic regression, and lasso. Each of these models was wrapped from the widely used machine learning library, scikit-learn.^7^

#### Model Module: Encapsulating DNNs

In DNNBrain, a DNN model is implemented as a neural network model from PyTorch. Each DNN model is a sequential container which holds the DNN architecture (i.e., connection pattern of units) and associated connection weights. The DNN model is equipped with a suite of methods that access attributes of the model and update states of the model. PyTorch has become the most popular DL framework because of its simplicity and ease of use in creating and deploying DL applications. At present, several well-known PyTorch DCNN models^12^ pretrained for different stimulus modalities have been adopted into DNNBrain, including AlexNet (Krizhevsky et al., 2012), VGG (Simonyan and Zisserman, 2015), GoogLeNet (Szegedy et al., 2015), and ResNet (He et al., 2016) for image classification; VGGish for audio classification (Hershey et al., 2017); and R3D for video classification (Tran et al., 2018).

#### Algorithm Module: Characterizing DNNs and Brains

The algorithm module defines various algorithms objects for exploring DNNs. An algorithm object contains a DNN model and corresponding methods that allow the study of specific properties of the model. Three types of algorithms are implemented in DNNBrain. The first type is the gradient descent algorithm for DNN model training, which is wrapped from PyTorch.^13^ The second type of algorithm comprises tools for extracting and summarizing the activation of a DNN model, such as principal component analysis (PCA) and clustering. The third type is made up of algorithms that visualize representations of a DNN, including discovering the top stimulus, mapping saliency features of a stimulus, and synthesizing the maximum activation stimulus for a specific DNN channel. Each algorithm takes a DNN model, as well as a stimulus object, as input.

#### Command Line Interface

At the application level, DNNBrain provides several workflows as command line interface, including those that access DNN representations, visualize DNN representations, evaluate the behavioral relevance of the representations, and map DNN representations to brains. Users can conveniently run commands to perform typical representation analysis on their data.

#### Extension of DNNBrain

Along with the modules and algorithms that have already been implemented in DNNBrain, the user can extend DNNBrain in the following ways. First, any PyTorch model can be easily wrapped into DNNBrain by inheriting DNN Class and overriding its few methods. Second, any linear or non-linear model can conveniently be introduced into DNNBrain as either an encoding/decoding model, as long as they have the same interface as the scikit-learn Classifier/Regression object. Finally, users can write their own scripts to develop customized pipelines by reusing the algorithms and dataset objects.

## Methods

### DNN Model: AlexNet

**Figure 4:**
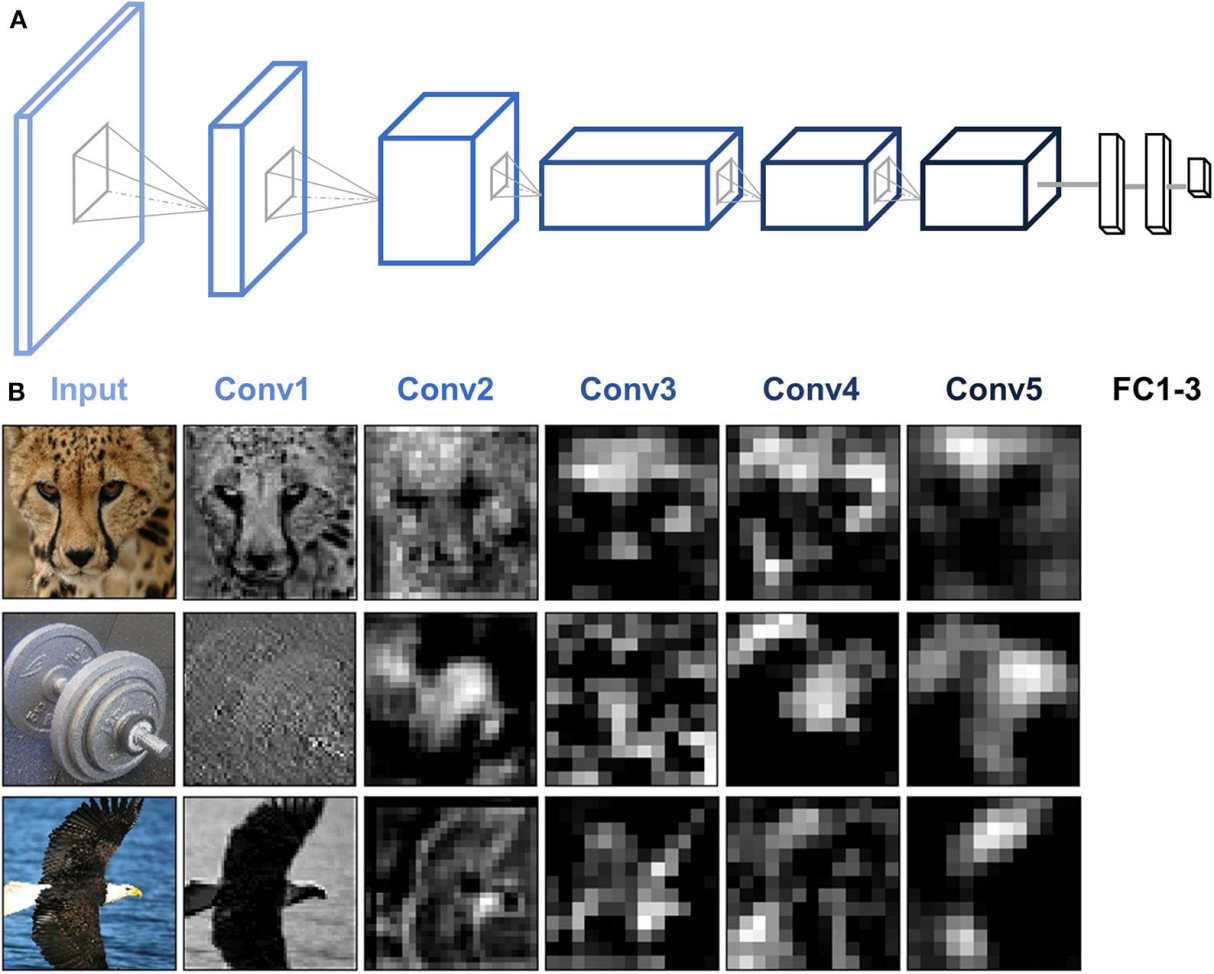
AlexNet architecture and activity patterns from example units. **(A)** AlexNet consists of five Conv layers followed by three FC layers. **(B)** The activation maps from each of the five Conv layers of AlexNet were extracted for three example images (cheetah, dumbbell, and bald eagle). Presented channels are those showing maximal mean activation for that example image within each of the five Conv layers.

AlexNet is used as an example to illustrate the functionality of DNNBrain. AlexNet is one of the most influential DCNNs. In the 2012 ImageNet challenge (Krizhevsky et al., 2012), it demonstrated for the first time that DCNNs can increase ImageNet classification accuracy by a significant stride. AlexNet is composed of five convolutional (Conv) layers and three fully connected (FC) layers that receive inputs from all units in the previous layer (Figure 4A). Each Conv layer is generally composed of a convolution, a rectified linear unit function (ReLU), and max pooling operations. These operations are repeatedly applied across the image. In this paper, when we refer to Conv layers, we mean the output after the convolution and ReLU operations.

Because AlexNet contains thousands of units in each layer, the dimension (i.e., the number of units) of the activation patterns from each layer was reduced to 100 via PCA to avoid the risk of overfitting the models in further analyses of DNN and brain representation.

### BOLD5000: Stimulus and Neuroimaging Data

BOLD5000 is a large-scale publicly available human functional MRI (fMRI) dataset in which four participants underwent slow event-related BOLD fMRI while viewing ~5,000 distinct images depicting real-world scenes (Chang et al., 2019). The stimulus images were drawn from the three most commonly used computer vision datasets: 1,000 hand-curated indoor and outdoor scene images from the Scene UNderstanding dataset (Xiao et al., 2010), 2,000 images of multiple objects from the Common Objects in Context dataset (Lin et al., 2014), and 1,916 images of mostly singular objects from the ImageNet dataset (Deng et al., 2009). Each image was presented for 1 s followed by a 9-s fixation cross. Functional MRI data were collected using a T2^*^-weighted gradient recalled echo planar imaging multi-band pulse sequence (In-plane resolution = 2 × 2 mm; 106 × 106 matrix size; 2 mm slice thickness, no gap; TR = 2,000 ms; TE = 30 ms; flip angle = 79°). The scale, diversity, and naturalness of the stimuli, combined with a slow event-related fMRI design, make BOLD5000 an ideal dataset to explore the DNNs and brain representations of a wide range of visual features and object categories. The raw fMRI data were preprocessed utilizing the fMRIPrep pipeline including motion correction, linear detrending, and spatial registration to native cortical surface via boundary-based registration (Esteban et al., 2019). No additional spatial or temporal filtering was applied. For a complete description of the experimental design, fMRI acquisition, and preprocessing pipeline, see Chang et al. (2019).

The preprocessed individual fMRI data were firstly transformed into 32k_fs_LR space using ciftify (Dickie et al., 2019). BOLD response maps for each image were then estimated from the fMRI data using the general linear model (GLM) from HCP Pipelines (Glasser et al., 2013). The response maps of each image were finally averaged across four subjects in the fsLR space and used for further analyses. Moreover, we constrained our analysis to the ventral temporal cortex (VTC), a critical region for object visual recognition. The VTC region was defined by merging the areas V8, FFC (fusiform face complex), PIT (posterior inferotemporal complex), VVC (ventral visual complex), and VMV (ventromedial visual areas) from HCP MMP 1.0 (Glasser et al., 2016). DNNBrain pipelines support both surface and volume data. Here, we preferred to use surface-based preprocessed data instead of volume-based preprocessed data because previous studies have shown that surface-based analysis can increase the specificity of cortical activation patterns (Van Essen et al., 1998; Brodoehl et al., 2020).

## Results

We demonstrated the functionality of DNNBrain on AlexNet and BOLD5000 dataset. Specifically, we accessed DNN activation of the images from BOLD5000, probed the category information represented in each DNN layer, mapped the DNN representations onto the brain, and visualized the DNN representations. We do not aim to illustrate the full functionalities that are available from DNNBrain, but rather to sketch out how DNNBrain can be easily used to examine DNN and brain representations in a realistic study. All the analyses were implemented in both API and CLI levels. The code can be found in the DNNBrain online documentation.^4^

### Scanning DNNs

To examine the artificial representations of DNNs, we needed to scan the DNN to obtain its neural activities, just as we scan the human brain using brain imaging equipment. DNNBrain provides both API and CLI to extract activation states for user-specified channels of a DNN. Figure 4 shows the activation patterns of three example images (cheetah, dumbbell, and bald eagle) from the channels of AlexNet which showed the maximal mean activation within each of the five Conv layers. The activation patterns revealed that DNN representations of the images became more abstract along the depth of the layers.

### Revealing Information Presented in DNN Layers

**Figure 5:**
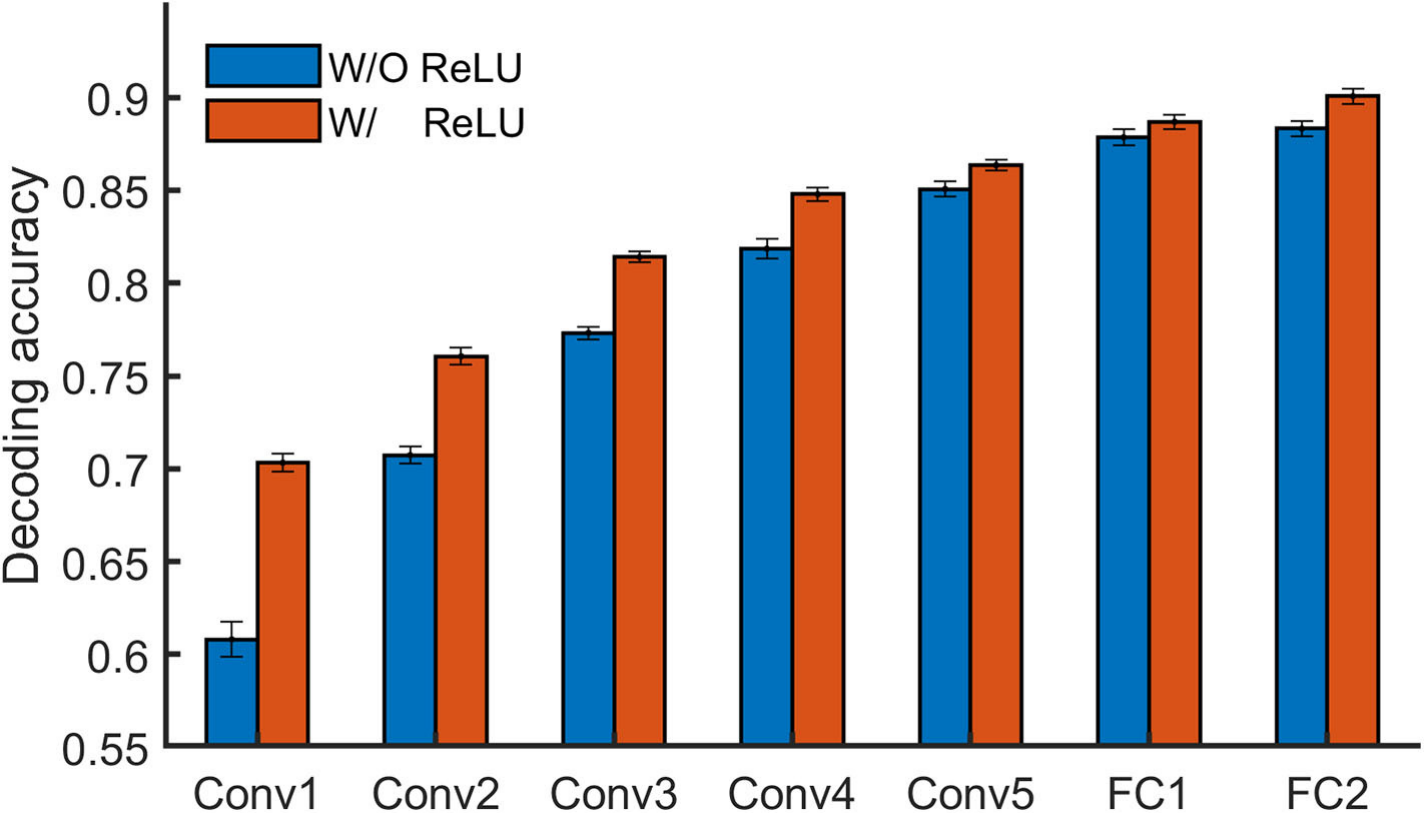
DNNBrain provides linear decoding models to probe the explicit representation contents of layers of interest in a DNN. On BOLD5000 stimuli, a logistic regression model revealed that the higher a layer is, the more animate information is encoded within it.

To learn whether specific stimuli attributes or behavioral performances are explicitly encoded in a certain layer of a DNN, one direct approach is to measure to what degree the representation from the layer is useful for decoding them. Linear decoding models (classifier or regression) were implemented in DNNBrain to enable this. Here, we manually sorted BOLD5000 stimulus images into binary categories (animate vs. inanimate) according to salient objects located in each image, and then examined how animate information is explicitly encoded in AlexNet. In total, 2,547 images were labeled as animate and 2,369 as inanimate. We trained a logistic regression model on the artificial representations to decode the stimulus category for each Conv layer of AlexNet. The accuracy of the model was evaluated with a 10-fold cross-validation. As shown in Figure 5, the classification accuracy progressed with the depth of Conv layers, indicating higher layers encoded more animate information than lower layers. Moreover, the ReLU operation within each Conv layer played a significant role in improving the representation capacity for animate information.

### Mapping Representations Between a DNN and the Brain

**Figure 6:**
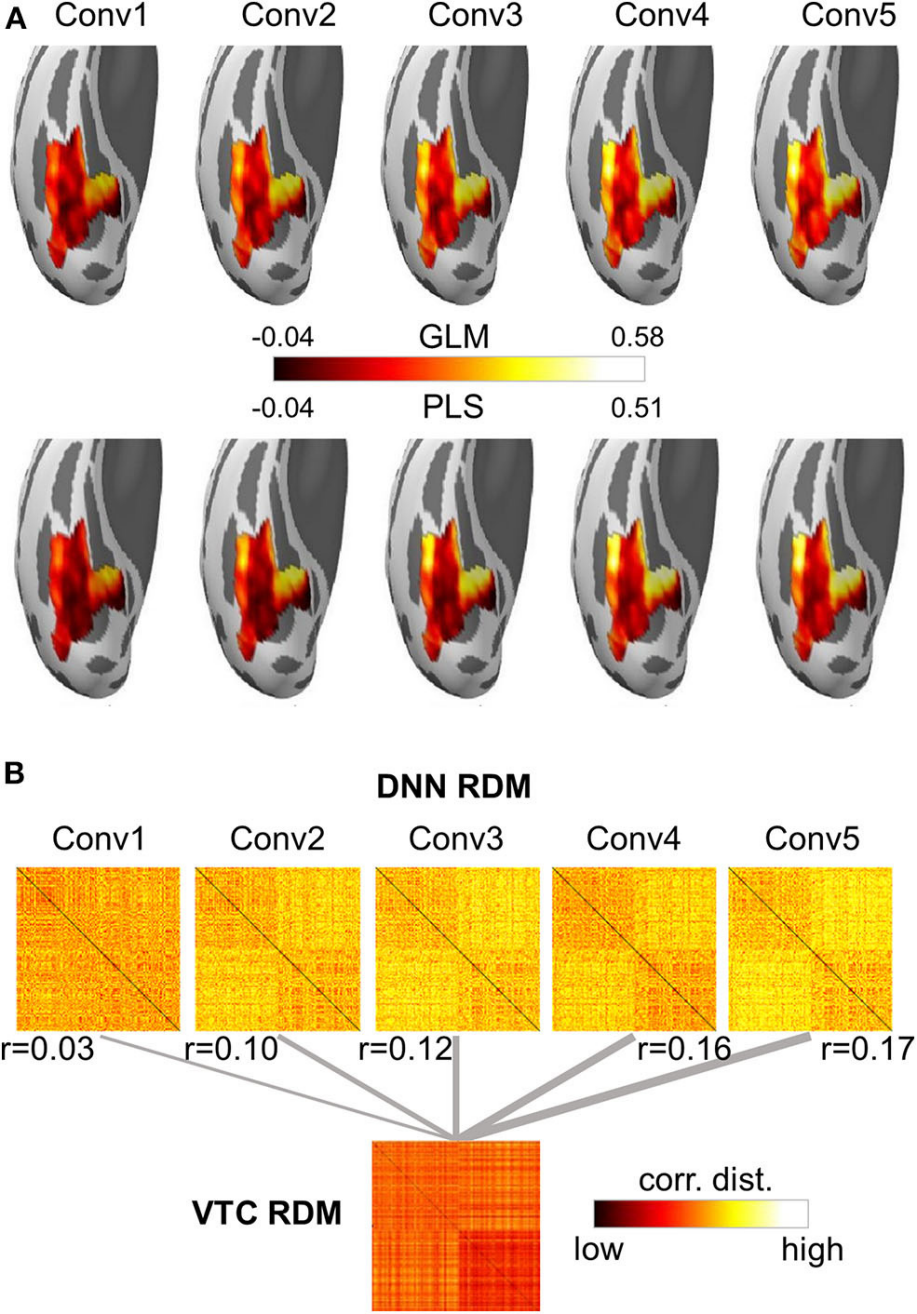
Both the encoding model and the representational similarity analysis are implemented in DNNBrain to help researchers to examine the correspondence between the DNN and brain representations. **(A)** Encoding accuracy maps from univariate GLM encoding models of predicting VTC BOLD responses using artificial representation from the Conv layers of AlexNet (top), and encoding accuracy maps from multivariate PLS encoding models of predicting VTC BOLD responses using artificial representation from the Conv layers of AlexNet (bottom). **(B)** RDMs for BOLD5000 stimuli computed on artificial representations from Conv layers of AlexNet and brain activation patterns from the human VTC. The representation distance between each pair of images was quantified as the correlation distance between their representations. The representational similarity between the DNN and the brain is further calculated as the Pearson correlation between their RDMs.

A growing body of literature is investigating the potential of DNNs to work as models of brain information processing. Several recent studies found that internal representations of object recognition DNNs provided the best current models of representations of visual images in the inferior temporal cortex of both humans and monkeys (for a recent review, see Lindsay, 2020). Here, we adopted the univariate encoding model, multivariate encoding model, and RSA on BOLD5000 dataset to map artificial representations from Conv layers of AlexNet to neural representations from the VTC of the brain. On the artificial representation from each Conv layer of AlexNet, a univariate GLM encoding model was constructed for each voxel within the VTC, and a multivariate PLS encoding model was built for the whole VTC. Encoding accuracy was evaluated with the Pearson correlation between the measured responses and the predicted responses from the encoding model using a 10-fold cross-validation procedure. For RSA, RDM was derived using the correlation distance between each pair of stimuli, and a Pearson correlation was used to measure the similarity between two representations. Four main findings were revealed (Figure 6). First, the encoding accuracy of the VTC gradually increased for the hierarchical layers of AlexNet, indicating that as the complexity of the visual representations increases along the DNN hierarchy, the representations become increasingly VTC-like. Second, the encoding accuracy varied greatly across voxels within the VTC for the artificial representations of each AlexNet layer, suggesting the VTC may organize in distinct functional modules, each preferring different kinds of features. Third, the univariate encoding model and the multivariate encoding model produced similar results, indicating that interactions between different voxels encode little representation information from each DNN Conv layer. Finally, RSA also showed results similar to those of encoding models, suggesting that the encoding model and RSA are likely to be equally useful for comparing representations from DNNs and brains.

### Visualizing Features From DNNs

**Figure 7:**
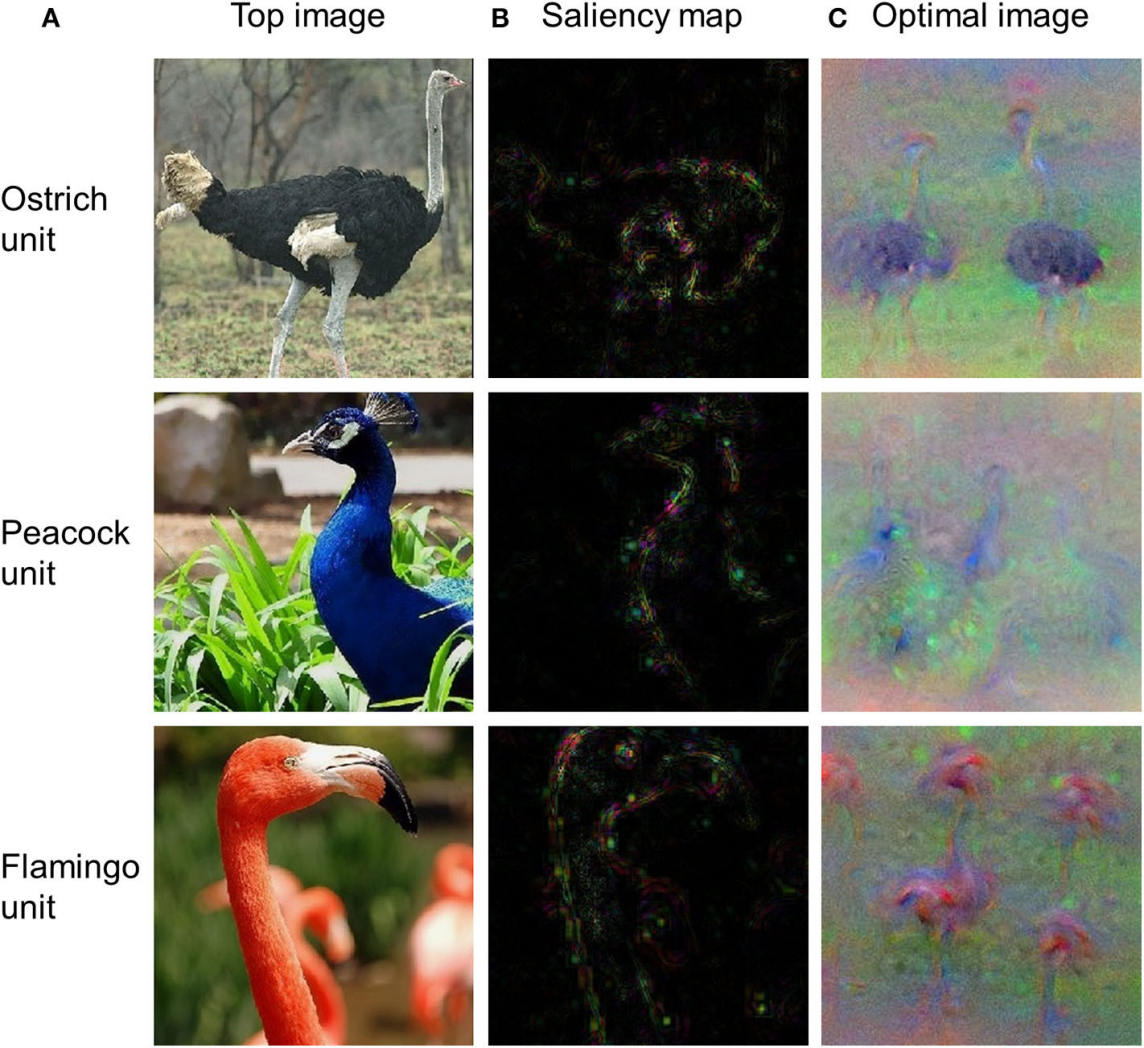
The top stimuli, saliency maps, and optimal images for three output units of AlexNet. **(A)** Top stimuli discovered from the BOLD5000 dataset. **(B)** Saliency maps computed for the top stimuli presented in **(A)**. **(C)** Optimal images synthesized from initial random noise guided by increasing the activation of corresponding neurons.

Visualization of critical features of a stimulus that cause the responses of an artificial neuron is central to the understanding of precisely what each neuron has learned. As an example, we used three DNN visualization approaches from DNNBrain (i.e., top stimulus, saliency map, and optimal stimulus) to visualize the preferred features for three output units of AlexNet (i.e., ostrich, peacock, and flamingo). The output units were selected as examples because produced features for them are easy to check (i.e., each unit corresponds to a unique category). These procedures essentially work for any unit in a DNN. As shown in Figure 7A, the top stimulus was correctly found from 4,916 BOLD5000 images for each of three units: every top stimulus contains the object in the correct category. Saliency maps highlight the pixels in the top stimuli that contribute most to the activation of the neurons (Figure 7B). Finally, the optimal images synthesized from initial random noise correctly produced objects of the corresponding category (Figure 7C). In summary, these three approaches are able to reveal the visual patterns that a neuron has learned on various levels and thus provide a qualitative guide to neural interpretations.

## Discussion

DNNBrain integrates well-established DNN software and brain imaging packages to enable researchers to conveniently map the representations of DNNs and brains, and examine their correspondences. DNN models provide a biologically plausible account of biological neural systems, and show great potential for generating novel insights into the neural mechanisms of brains. On the other hand, experimental paradigms from cognitive neuroscience provide powerful approaches to pry open the black boxes of DNNs. DNNBrain, as a toolbox that is specifically tailored toward mapping the representations of DNNs and brains, has good potential to accelerate the merge of these two trends.

There are some issues that we would like to target in future development. First, DNNBrain integrates many of the currently most popular pretrained DCNN models. With the advance of the interplay between neuroscience and DNN communities, new DNN models are constantly emerging, and will be included into future iterations of DNNBrain. For example, generative adversarial networks could be introduced into DNNBrain to help users reconstruct external stimuli (Shen et al., 2019; VanRullen and Reddy, 2019) or synthesize preferred images for either neurons or brain areas (Ponce et al., 2019). Second, DNNBrain, up until now, only supports DNN models from PyTorch, which limits the study of DNNs constructed under other frameworks. We would like to put significant effort toward integrating other DNN frameworks into DNNBrain, especially TensorFlow. Third, only fMRI data are currently well-supported in DNNBrain. The magnetoencephalography (MEG), electroencephalography (EEG), multiunit recordings, and local field potentials can capture the temporal dynamics of neural representations which fMRI cannot provide. Support for these modalities is forthcoming according to recently published data standardization of electrophysiology (Niso et al., 2018; Pernet et al., 2019). Finally, DNNBrain mainly supports the exploration of pretrained DNN models, trained on large-scale external stimuli. It would be a good idea in the future to equip DNNBrain with tools that fuse brain activities and external tasks/stimuli to create DNN models that more closely resemble the human brain. Recent advances demonstrate that brain representations provide additional and efficient constraints on DNN constructions (McClure and Kriegeskorte, 2016; Fong et al., 2018). The brain has acquired a robust representation that generalizes across many tasks. As a result, while training DNNs to solve behavioral tasks, co-training DNNs to match the brain's latent representations observed from massive neural recordings will move the representation of DNNs toward these neural representations, and make them more closely resemble the human brain.

## Data Availability Statement

DNNBrain is freely available via github.^3^ The code and data used in this article is available from readthedocs^4^ and OSF^14^, respectively. Further inquiries can be directed to the corresponding authors.

## Ethics Statement

All procedures followed the principles in the Declaration of Helsinki. Participants all provided written informed consent. The experimental MRI protocols for BOLD5000 were approved by the Institutional Review Board (IRB) of Carnegie Mellon University. The reported analyses in this study were approved by the IRB of the Beijing Normal University.

## Author Contributions

XC, MZ, ZG, WX, XL, TH, and ZZ developed the toolbox and designed the validations. XC, ZZ, and JL wrote the paper. MZ, ZG, WX, XL, and TH revised and approved the paper. All authors contributed to the article and approved the submitted version.

## Conflict of Interest

The authors declare that the research was conducted in the absence of any commercial or financial relationships that could be construed as a potential conflict of interest.

## Footnotes

^1^https://pytorch.org

^2^https://www.tensorflow.org

^3^http://github.com/BNUCNL/dnnbrain

^4^http://dnnbrain.readthedocs.io

^5^https://numpy.org

^6^https://www.scipy.org

^7^https://scikit-learn.org

^8^http://pythonware.com/products/pil

^9^https://nipy.org

^10^https://fmriprep.org

^11^https://nipy.org/nibabel

^12^https://github.com/pytorch/vision

^13^https://pytorch.org/docs/stable/optim.html

^14^https://osf.io/hy5m7
